# Tangles, Toxicity, and Tau Secretion in AD – New Approaches to a Vexing Problem

**DOI:** 10.3389/fneur.2013.00160

**Published:** 2013-10-21

**Authors:** Kerry L. Gendreau, Garth F. Hall

**Affiliations:** ^1^Department of Biological Sciences, University of Massachusetts Lowell, Lowell, MA, USA

**Keywords:** tau oligomerization, tau toxicity, tau secretion, interneuronal lesion spread, exosome

## Abstract

When the microtubule (MT)-associated protein tau is not bound to axonal MTs, it becomes hyperphosphorylated and vulnerable to proteolytic cleavage and other changes typically seen in the hallmark tau deposits (neurofibrillary tangles) of tau-associated neurodegenerative diseases (tauopathies). Neurofibrillary tangle formation is preceded by tau oligomerization and accompanied by covalent crosslinking and cytotoxicity, making tangle cytopathogenesis a natural central focus of studies directed at understanding the role of tau in neurodegenerative disease. Recent studies suggest that the formation of tau oligomers may be more closely related to tau neurotoxicity than the presence of the tangles themselves. It has also become increasingly clear that tau pathobiology involves a wide variety of other cellular abnormalities including a disruption of autophagy, vesicle trafficking mechanisms, axoplasmic transport, neuronal polarity, and even the secretion of tau, which is normally a cytosolic protein, to the extracellular space. In this review, we discuss tau misprocessing, toxicity and secretion in the context of normal tau functions in developing and mature neurons. We also compare tau cytopathology to that of other aggregation-prone proteins involved in neurodegeneration (alpha synuclein, prion protein, and APP). Finally, we consider potential mechanisms of intra- and interneuronal tau lesion spreading, an area of particular recent interest.

## Overview

Cytotoxicity associated with the accumulation of abnormal protein aggregates has emerged as a central common mechanism underlying human neurodegenerative disease. Neurons are unique among differentiated cell types in that they do not re-enter the cell cycle and thus cannot use mitosis as a method for clearing abnormally aggregated proteins. As a result, they are inherently vulnerable to disruption of protein turnover mechanisms such as the ubiquitin/proteasome pathway and autophagy, especially in aged individuals. The abnormal turnover of aggregation-prone proteins such as alpha synuclein (SNCA), prion protein (PrP), amyloid beta (Ab) peptide, and tau are thus key factors in most (95%) of the neurodegenerative diseases that affect humans. Protein aggregation is typically accompanied and potentiated by abnormal phosphorylation, ubiquitination, covalent crosslinking, and the abnormal activation of autolytic proteases (1–6). A common feature of such proteins is an “intrinsically disordered” structure (4), in which the normal conformation can be readily changed into a beta-sheet rich structure with high aggregation propensity (6). Moreover, conditions in which SNCA (Parkinson’s disease, Lewy Body Dementia), tau (Corticobasal Degeneration, Pick’s disease, Frontotemporal Dementia), and PrP [Creutzfeldt–Jakob disease (CJD), Gerstmann–Straussler–Scheinker disease] form abnormal aggregates typically show overlapping neuropathology, suggesting that synergistic interactions may occur between these proteins in each of these conditions (7). The aggregation of the microtubule (MT)-associated protein tau, which is heat stable and normally exhibits a random coil conformation in aqueous solution, plays a central role in the neurodegeneration seen in Alzheimer’s disease and non-Alzheimer’s tauopathies that form the core of the paired helical filament (PHF) (8, 9). PHFs and related filamentous aggregates such as straight and ribbon-like filaments (10) in turn make up the hallmark tau lesions [neurodegenerative diseases (NFTs), Pick bodies, etc.] seen in Alzheimer’s disease and other tauopathies. However it is likely that at least some of the characteristic features that distinguish tauopathies from other neurodegenerative syndromes have roots in specific normal cellular functions of tau.

### MT and non-MT associated functions of tau

Tau is expressed from a single gene on chromosome 17 and is alternatively spliced to yield six different isoforms in the adult central nervous system (CNS). Each of these contains a C-terminal microtubule binding domain (MTBR) consisting of three or four tandem repeat motifs. The best understood “normal” function of tau in mature neurons involves its binding to and stabilizing axonal MTs via the MTBR. Tau belongs to a family of microtubule associated proteins (MAPs) that includes other neuronal proteins such as MAP1A, MAP1B, and MAP2, and also non-neuronally expressed members (MAP4). Each of these proteins contains a conserved region in and around the MTBR that suggests a common origin via gene duplication. The poor conservation of areas outside of the MTBR in both mammalian tau family molecules and in tau-like proteins in various vertebrate and invertebrate species suggests that they may play species-specific functions.

The regulation of tau-MT binding has been heavily studied and is now well established. Tau-MT binding associated with MT stabilization is mediated by the phosphorylation of serine and threonine residues at sites immediately adjacent to and within the MTBR by a wide variety of kinases [Ref. (11) for a good review]. Phosphorylation of the regions flanking the MTBR produce a stoichiometrically graded reduction in the affinity of tau for MTs, whereas phosphorylation at specific sites within the MTBR (Ser262 and Ser356) abolish virtually all tau-MT interactions (12). Subtleties of tau-MT binding appear to be particularly dependent on the phosphorylation pattern of proline-associated serine and threonine resides on the N-terminal side of the MTBR by proline-directed kinases (e.g., CDK5, MAP kinase 1, GSK3b).

It has become clear that tau has functions in addition to axonal MT stabilization in both mature and developing neurons that involve alternative binding partners for the MTBR (e.g., actin- and actin-associated proteins, heparin sulfate proteoglycan) and/or other parts of the tau molecule, such as the amino terminal projection domain (13). These functions include the integration of cellular cytoskeletal functions with interneuronal signaling pathways. Important developmental functions of tau include various aspects of axonogenesis, such as the establishment of axonal identity (i.e., neuronal polarization) (14) and the subsequent outgrowth (15) and myelination of developing axons. Each of these developmental functions involves MTBR interactions with the subcortical actin network and plasma membrane (16, 17), via either the MTBR itself [actin – Ref. (18)] or via the N-terminal projection domain, which interacts with Src family non-receptor tyrosine kinases such as fyn (13, 19, 20).

### Disease-associated tau modifications are correlated with their dissociation from MTs

#### Tau hyperphosphorylation

A key alteration that is associated with NFT formation is the phosphorylation of multiple serine and threonine residues in and around the MTBR that normally regulate tau binding to MTs (hyperphosphorylation). Hyperphosphorylation reversibly decreases the affinity of tau for MTs (21, 22) and is consistently seen in PHFs isolated from AD and non-AD tauopathy brains even at early stages in the development of disease (23–27). Hyperphosphorylated tau defined as containing 10 or more moles of phosphate per tau molecule (28) isolated from AD brains has been shown to be capable of self-assembly *in vitro* (29), suggesting that tau hyperphosphorylation may directly induce aggregate formation as well as altering the normal functions of tau. Tau mutations associated with frontotemporal dementia with Parkinsonism linked to chromosome 17 (FTDP-17) increase the rate and amount of tau phosphorylation and decrease the number of phosphate groups required for aggregation (11, 28). Although clearly associated with PHF formation, hyperphosphorylation may not be a prerequisite for tau aggregation, since tau constructs containing only the MTBRs are able to form filaments in the unphosphorylated state (29, 30). However, dephosphorylation prevents the self-assembly of full-length isoforms, suggesting that the N- and extreme C-terminal regions of tau inhibit this (31, 32). Curiously, phosphorylation of tau at certain residues (Ser262 and Ser 214) that decrease tau-MT affinity act to prevent formation of PHFs (33). This, together with multiple reports of phosphorylation state-contingent kinase specificity for individual sites in the flanking domains, suggests that regulation of both tau:tubulin and tau:tau affinity by phosphorylation is highly subtle and remains incompletely understood.

#### Tau truncation

The evolution of tangle-intrinsic tau from full-length isoforms to MTBR-only fragments (34) suggests that proteolysis at both the C and N termini of tau may play a significant role in NFT formation. This is directly supported by a number of studies of tau filament formation. Temporal analysis of filament formation in AD brains shows that tau misfolding [recognized by the Alz50 antibody (35)] precedes initial truncation at D421, with subsequent truncation at E391 appearing at later stages of the disease (34). Truncated C-terminal tau fragments can act as nucleation seeds *in vitro*, sequestering full-length tau in both mutant and wild-type forms (11). Such nucleation may be related to conformation specific tau:tau interactions that have recently been proposed to mediate the intercellular propagation of neurofibrillary lesions (36, 37). Truncation of tau at E391 has been identified within the core of PHFs (38) and at D421 in the brains of AD patients (39). The presence of the C terminal has an inhibitory effect on polymerization of full-length tau (31) and C-terminal truncation accelerates tau filament formation *in vitro* (40–42). A study using cells expressing the MTBR of tau containing an FTDP-17 mutation demonstrated that proteolysis of the N and C regions flanking the MTBR produced aggregation-prone fragments capable of seeding further aggregation, while blocking N-terminal truncation of this fragment prevented C-terminal proteolysis, suggesting that this region (near K257) may act to shield downstream residues (11). It should be noted that although C-terminal truncation is sufficient to cause tau aggregation in cellular models, additional mechanisms might drive filament formation in diseased neurons. Tangle-bearing neurons in a transgenic mouse model contained little D421-cleaved tau (43), suggesting that multiple pathways to aggregate formation are likely active in neurons affected by tauopathy. This is consistent with the presence of multiple types of lesions in the various forms of non-AD tauopathy and in AD brain, where neuropil threads (NTs) occur with or perhaps even before the onset of NFT formation (44) and with the characteristic differences in neurofilament content and tau conformation seen in NTs (45) and Pick bodies (10, 46) versus NFTs.

#### Other variables affecting tau aggregation

In addition to phosphorylation and truncation, there are a number of factors that appear to modulate tau aggregate formation, some of which are associated with tauopathy cytopathogenesis. Foremost among these are the intronic and exonic point and deletion mutations in and around the MTBR that cause familial non-AD tauopathies [reviewed in Ref. (47)]. The restriction of such mutations to the MTBR and their ability to drive the neurofibrillary pathogenesis of presenile tauopathies constitutes some of the strongest evidence for the importance of tau aggregation in human disease. Polyanions such as heparan sulfate proteoglycan (HSPG) are abnormally distributed in pre-tangle and tangle-bearing neurons and can catalyze the formation of straight filaments and PHFs *in vitro* (48, 49) regardless of the phosphorylation state of tau (30, 50, 51). Specific isoforms of tau also differ in their ability to form filaments *in vitro*. Different ratios of three and four repeat (3R and 4R) tau isoforms are characteristic of tau aggregates found in specific tauopathies (26, 52) and the presence of intronic mutations interfering with the splicing of exon 10 in patients with FTDP-17 (30, 53) confirms that the resulting change in the ratio of 3R:4R tau is sufficient to drive tau filamentation and neurodegeneration (54). Three and four repeat isoforms differ in characteristics that may affect their participation in NFT formation, including the ability to form disulfide bridges (55) and their relative affinity for MTs and fyn kinase (56). The presence of 3R tau may reduce the tendency of 4R tau to form filaments (57), possibly by interfering with disulfide bond formation. Finally, the larger cellular and genetic context is likely to have a significant bearing on the tendency of tau to aggregate into NFTs. Early cellular changes involving lysosomal and autophagy pathway abnormalities may either result from or modulate tau aggregate formation (58, 59). Genetic factors such as the H1 haplotype, which affects the overall expression levels of tau and the splicing of exon 10 and possibly exons 2 and 3 (60), and the presence of ApoE4 allele have been reported to be associated with high disease incidence (61). Additional genetic factors that affect tau aggregation and thus disease propensity include interactions with elements on chromosome 21 that affect tau splicing and the function of non-APP proteins such as DYRK1A (53, 62) as well as APP itself (63). Overall, tau interactions with non-MT elements via its MTBR and in particular, tau:tau interactions associated with aggregation and their involvement in tau-mediated neurodegeneration have been a subject of intensive investigation. However, while a great deal is now known about both the mechanisms of tau aggregation and the circumstances of tau modifications associated with aggregate formation, it remains unclear exactly how each of these elements contributes to tau cytopathogenesis. This uncertainty has been exacerbated by the identification of tau toxicity mechanisms that are not associated with the MTBR but which may also play important roles in neurodegeneration.

### Reactivation of developmental tau functions in neurodegeneration

While tau aggregation is clearly a central factor in tauopathy pathogenesis, the role tau plays in the development of axonal identity and other aspects of axonogenesis has increasingly been linked to neurodegenerative disease mechanisms. These include the appearance of cell cycle markers (64) and ectopically sprouting axonlike processes (NTs) emerging from the dendrites (45, 65–67) during the development of neurofibrillary lesions in neurodegenerative tauopathies. Dendritic NTs in particular suggest that tau functions in the development of axonal identity may play a role in the early stages of AD where they may reflect damage to mechanisms that maintain the terminally differentiated neuronal state (68, 69). The “fetal” (3R0N) tau isoform is the shortest three repeat tau isoform with the lowest binding affinity for MTs (30), possibly reflecting the far greater importance of tau N-terminal interactions and functions during development relative to the role tau plays in the mature CNS. A key tau interactor during development is the non-receptor tyrosine kinase fyn, which plays a critical role in axonal outgrowth and myelination (15, 70). Interactions between the tau N terminus and signaling molecules such as fyn typically occur in the actin-rich cortical cytoskeleton largely in the absence of MTs and are necessary for functions such as growth cone motility (16). Fyn, like tau, localizes to NFTs (20, 71) and is essential to the development and possibly the propagation of Abeta-mediated toxicity in mouse models of AD (72). Fyn-mediated interactions with tau play a role in the localization of a small amount of tau to the plasma membrane (73), particularly at dendritic loci (74) where it is involved in synaptic functions associated with learning and memory (75–77). Abnormal interactions of tau with fyn kinase increasingly appear to play a critical role in membrane-associate tau dysfunction especially via the generation of synaptotoxic species of Abeta from APP. This occurs in the context of fyn-mediated phosphorylation of APP in early endosomes (78) and in turn exacerbates both tau localization to rafts and tau phosphorylation by fyn (79). Such correlations suggest that tau mislocalization to dendrites and the generation of abnormal amounts of Abeta may interact synergistically to produce both cytotoxicity and abnormal developmental events such as NT growth and cell cycle re-entry (80) in AD neuropathogenesis.

### Interneuronal aspects of tauopathy

Tau interactions with lipid raft proteins such as fyn and (possibly) APP may be centrally important in two recently emerging interneuronal aspects of tau pathobiology: (a) tau secretion and interneuronal transfer via unconventional mechanisms and (b) tau:tau interactions involved in templated misfolding and “prionlike” lesion spreading mechanisms. Membrane localization favors tau oligomerization, which increasingly appears to be a key event in at least some forms of tau toxicity. Membrane-associated tau functions mediated by its N terminus appear to be linked to the diversion of tau from the cytosol to membrane-bound vesicles, in particular those associated with the trans-Golgi network and the autophagosome-lysosome pathway (81). As a consequence, it is likely that both cellular and molecular aspects of interneuronal lesion spreading have their roots in membrane-associated tau misprocessing mechanisms.

#### Selective neuronal vulnerability to tau toxicity

Ever since it became apparent in the mid-1980s that Alzheimer’s disease is a common neurodegenerative condition of the elderly as opposed to a relatively rare familial syndrome, it has been clear that the number and distribution of NFTs is strongly correlated with progressive cognitive loss (82, 83). There are two possible mechanisms that might account for disease-associated stereotyped patterns of lesion development with increasing disease severity. One possibility is that loci that are affected early in the disease sequence are more vulnerable to the mechanisms underlying tau-induced degeneration at the cellular level. A hierarchy of vulnerability to tau toxicity might then result in a stereotyped sequence of lesion development over time. One characteristic shared by brain regions that develop early tau lesions in AD is synaptic plasticity; highly plastic cortical pyramidal neurons such as those of the hippocampus are inherently vulnerable to excitotoxic insults by virtue of their glutamatergic pharmacology and the prevalence of LTP-associated plasticity mechanisms (84). Selective vulnerability may also be acquired via injury, as with the greatly increased risk posed by antecedent head trauma (85–87). It seems increasingly likely that both intrinsic and extrinsic selective vulnerability factors interact with lesion spreading mechanisms associated with synaptic connectivity patterns (discussed at length below) to produce the variety of clinical syndromes associated with neurofibrillary degeneration.

#### Interneuronal movement of misprocessed tau

For many years, the interconnectedness of affected regions and the highly stereotyped, disease-specific pattern of neurofibrillary lesion spread within the brain in AD and non-AD tauopathies has suggested that the actual interneuronal transfer of a toxic factor must be involved in the progression of neurodegenerative tauopathies (88–91). The actual movement of toxic tau species between neurons is now thought by many to be the primary mechanism mediating the progressive appearance of tau pathology and clinical dysfunction in tauopathy, including those associated with repeated head injury (87). In particular, the templated misfolding mechanism that mediates the infectivity of prion diseases such as CJD is increasingly invoked as a model for the interneuronal transfer of tau-mediated toxicity (36, 92–97), especially in murine transgenic models of tauopathy (98–100). Evidence that different abnormal conformations of PrP may in fact define different prion-mediated diseases (101, 102) raises the possibility that very different clinical presentations and neuronal vulnerabilities could be attributable to molecular level differences in a single protein. However, the study of tau as a “tauon” [a term for a tau species that spreads toxicity via templated misfolding coined by Novak et al. (93)] has been conducted largely in the absence of a cellular context for the actual transfer of tau between neurons, making it difficult to connect the molecular mechanisms involved in protein templating to specific cellular events and mechanisms associated with tau cytopathology. In particular, while the secretion (103–105) and uptake (92) of tau itself in cellular models has been demonstrated and linked to the elevated tau levels in early AD (106), it is unclear how secretion is related to the spreading of neurofibrillary lesions and whether that occurs via an oligomer-associated mechanism, such as templated misfolding or ionophore formation (107, 108), or via some mechanism that does not require the tau MTBR at all (109–112). The subtle but important questions that remain about the relationships between the development of NFTs, toxic oligomer formation, templated misfolding, N-terminal tau toxicity, and the actual cellular mechanisms responsible for secretion and uptake of misprocessed tau species will be explored at greater length below.

## Current Foci of Tauopathy Research

### The biogenesis of neurofibrillary tangles

The development of NFTs, the characteristic tau lesions that develop in cortical pyramidal neurons during the course of AD, has been relatively well characterized but is still incompletely understood. NFT development begins with the abnormal phosphorylation of tau at multiple sites in and around the MTBR, accompanied by abnormal somatodendritic tau accumulation resulting in the appearance of pre-fibrillary phosphorylated punctate deposits in the cell body and dendrites of affected neurons (24, 113). These eventually form condensed fibrillar deposits near the nucleus and near dendritic branch points, displacing normal cytoskeletal elements such as MTs (113, 114). Over time, these fill most of the cell and take on a characteristic flame-shaped appearance. Eventually, the neuron containing the NFT dies and the NFT remains as a “tombstone” lesion or “ghost” tangle.

While all tau isomers and cleavage fragments that contain the MTBR appear to be capable of forming filaments, their precise morphology may vary considerably; they can appear as straight, ribbon-like, or any of a variety of PHF-like structures, all of which are reproducible under *in vitro* conditions (10, 46, 115). Moreover, the wide variety of tau modifications that affect aggregation suggest that considerable subtlety exists in the mechanisms responsible for tau assembly. Such factors include the presence of tauopathy-inducing point mutations (116, 117) the presence/absence of the C-terminal (31) or N-terminal (10, 32) domains, and variations in the experimental conditions used (118). The fact that the range of filaments and aggregate types seen with human tauopathies can be reproduced in sporadic tauopathy syndromes without benefit of tau mutations indicate that additional relevant variables to tau aggregate form come from cellular factors, such as the inclusion of NFs (Pick bodies) or internal membrane elements (granulovacuolar degeneration) or even the shape of the host cell. Understanding how molecular and cellular factors interact to produce a specific type of aggregate can thus be seen as an index of our overall grasp of tauopathy pathogenesis.

#### Direct formation of NFTs from cytosolic tau

A key unresolved issue in NFT formation is the cellular context in which NFTs form during the course of neurodegenerative disease. In particular, it is unclear whether NFTs form as a result of cytosolic or even MT-templated interactions between tau species or whether NFT formation requires additional cellular elements, such as membrane-bound vesicles, to occur. The most straightforward hypothesis of NFT generation and growth is direct assembly from free tau monomers and/or oligomers in the cytosol. The ability of tau filaments to form *in vitro* by MT-templated mechanisms (119), and the ability of tauopathy mutations to actively disrupt MT networks (116) as well as favor filament formation (117, 120) are all consistent with the idea that tau aggregate formation, eventually leading to NFTs, begins immediately upon dissociation from MTs or even by conformational changes that occur in MT-bound tau. For instance, tau oligomerization has been observed to occur on the surface of MTs (119), which might then generate cytosolic tau oligomers capable of directly seeding NFT growth. This seems to account directly for the granular pre-fibrillary deposits that constitute the earliest visible stage of NFT formation (121, 122) and is consistent with *in vitro* experiments demonstrating that tau filament assembly resulting in NFT formation is potentiated by tau interactions with fatty acids and/or polyanionic molecules such as heparin sulfate proteoglycans and RNA (30, 50, 123, 124). Moreover, electron and atomic force microscopy studies have revealed numerous intermediates in tau fibril formation, including abnormally folded monomers capable of aggregation (125) and granular tau aggregates that may be formed from these monomers (121, 122). All of these observations are consistent with the direct formation of NFTs from cytosolic tau oligomers and this mechanism is therefore widely assumed to account for the biogenesis of most, if not all, NFTs.

#### Does vesicle-associated tau contribute to NFT formation?

**Figure 1:**
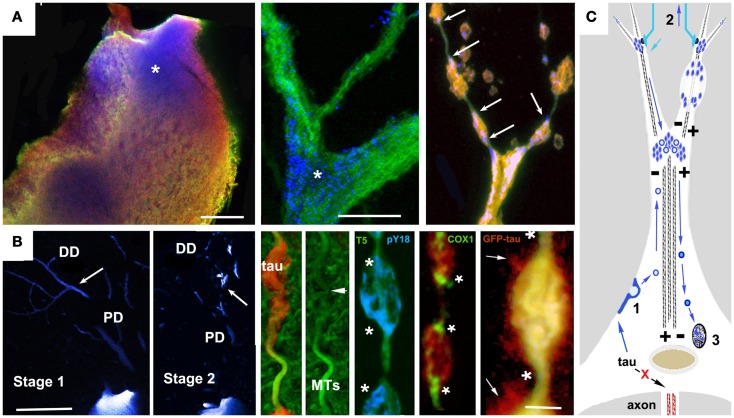
**Accumulation of vesicular tau in ABC dendrites and at dendritic branch points causes local transport failure, MT loss, and localized secretion from dendrites**. High resolution confocal imaging of somatodendritic tau accumulation in the lamprey tauopathy model suggests a cellular mechanism to account for the relationship between localized tau toxicity, somatodendritic MT loss, and the pattern of NFT evolution in pyramidal neurons as described by Braak et al. (113), Braak and Braak (137), and Blazquez-Llorca et al. (139). **(A)** Cell body (left) and dendritic branch point (center) of a lamprey ABC expressing full-length 4R0N human tau bearing the P301L tauopathy mutation before the onset of tau-induced degeneration. Tau is triple labeled: MT associated (Tau5, green channel), MT dissociated (9G3, blue channel), and total tau (GFP epitope tag red channel). Tau phosphorylated at Y18 (pY18 tau or 9G3 positive MT dissociated tau) is accumulating at the base of large dendrites and at branch points, a pattern typical of MT-transported vesicles (asterisks). The rightmost panel shows pY18 tau accumulating at either end of dendritic varicosities (arrows). **(B)** Left panels show pY18 distribution in non-degenerating (Stage 1) and degenerating (Stage 2) ABC dendrites. Distally transported tau is distributed throughout distal (but not proximal) dendrites in non-degenerating cells, but becomes localized to dendrite branch points and varicosities with the onset of degeneration [arrows – see Refs (81, 165)]. Center and right: Dendritic beading is caused by the localized failure of MT mediated transport, resulting in the accumulation of pY18 (fyn phosphorylated) tau associated with vesicles and membrane-bound organelles. The accumulation of mitochondria (COX2 label) is particularly well marked. With the onset of dendritic degeneration, total and pY18+ tau accumulates at each end and eventually in the center of dendritic varicosities in what appear to be MT-transported vesicles. The localized secretion occurring in the vicinity of such deposits suggests that tau-bearing vesicles first destabilize the MTs responsible for their transport, accumulate in the resulting varicosities and are then secreted. While the mechanism responsible for this has not been demonstrated directly, the concomitant loss of MTs and localized secretion suggests a Ca++ flux mediated mechanism. **(C)** A model for vesicle-associated tau in NFT formation and cytodegeneration. Failure of tau to become axonally localized and bind axonal MTs results in actin association and endocytosis (1). Tau-bearing vesicles are transported both distally and proximally on dendritic MTs, accumulating at dendritic branch points (where MT polarity patterns favor localized cargo accumulation Aronov 01) and near synaptic terminals (2), where it may become locally toxic possibly via interacting with Abeta in synapse-associated endosomes, resulting in structural failure of dendrites (top right) and uptake by afferents resulting in retrograde trans-synaptic movement. Synaptic activity may also result in the centripetal transport of tau-bearing vesicles to the Golgi apparatus (3) where it may modulate NFT formation. Scale bars: **(A)**: 20 μ, **(B)** 100 μ (left), 5 μ (right).

**Figure 2:**
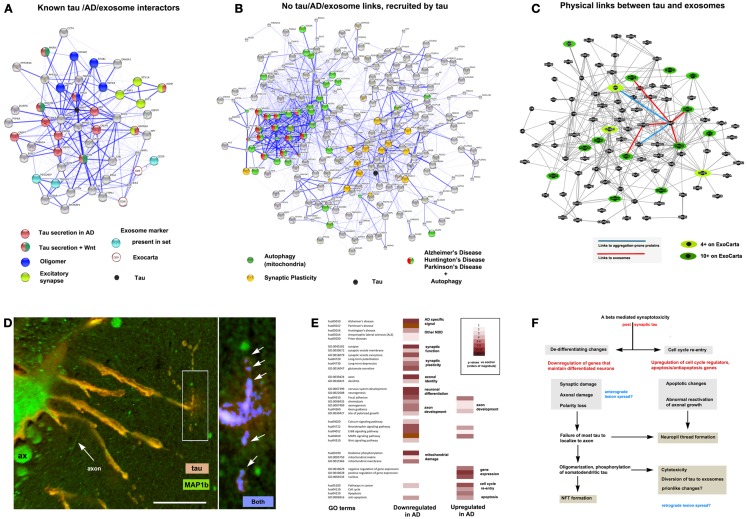
**The context of exosomal tau secretion and transneuronal neurodegeneration**. **(A)** Connectivity diagrams summarizing proteomic analysis of the exosomal proteome associated with tau overexpression from neuroblastoma cell cultures (106) using the String online dataset (104). GO term analysis shows that exosomal tau secretion involves factors with known links to tau misprocessing (APP, oligomerization, Wnt pathway) and also suggests the involvement of mechanisms with less established and no apparent links to tau, AD, and exosomes. Of the ∼660 proteins identified, 50 were both present on the AlzGene list (http://www.alzgene.org/) of 616 AD related proteins and had clear links (0.4 confidence or more (104) to tau and/or exosomal markers (CD9 and CD81 were not present in our set, but were part of the probe query) – see ExoCarta (239) via the String 9.05 connectivity algorithm. Note the strong linkage to tau (MAPT – shown as a black circle for reference). **(B)** Another group (right diagram) consisted of internally connected proteins that did not have clear functional links to tau on String (confidence <0.4 or not detected). These nonetheless had significant signals for AD, PD, and HD that were tightly linked to mitochondrial markers, suggesting the association of abnormal autophagy with tau secretion, and with synaptic plasticity (LTP, LDP). All terms shown reflect significant enrichment (*p* < 0.001) by the String algorithm. **(C)** Connectivity diagram from GeneMANIA (http://www.genemania.org) showing physical interactions between proteins isolated from exosomes using the same set as shown in **(A)**. In order to identify proteins typically localized to exosomes, we scored each protein for the number of times it appeared in the nearly 70 different mammalian exosomal datasets at ExoCarta. Links between tau and highly exosome associate proteins (dark green icons), mildly exosome associated (light green icons), and aggregation-prone proteins such as SNCA and APP (Abeta) are shown as well to illustrate the plausibility of tau diversion to exosomes in the context of these co-purified proteins. **(D)** Expression of 4R0N tau in lamprey ABCs produces dendritic localization of tau (red channel), dendritic degeneration, and localized focal secretion (left box, orange label) as shown in a confocal micrograph after 10 days of expression (81). Immunolabel for LC3/MAP1b (green channel) shows cytoskeletal localization in non-transfected giant axons (seen in cross section – ax), and in circumferential axons (arrow axon). One of the latter has taken up secreted tau shown at higher magnification (right) and is exhibiting toxicity in the form of varicosities (arrows) similar to those shown in ABC dendrites (dendrite, center). Highly co-localized tau (blue channel) shows cleaved, autophagosome-associated LC3II associated with tau (arrows) in a pattern similar to that seen for dendritic mitochondria in Figure 1B. Scale bar: 50 μ (left), 10 μ (right). **(E)** Consensus sets of 1575 downregulated and 1383 upregulated proteins in both LOAD (238) and early onset FAD (237). **(F)** Schematic outlining a hypothesis of AD cytopathogenesis that accounts for tau associated exosomal secretion and downregulation of synaptic and axonal proteins in AD (red) in the context of current knowledge. Likely triggers for tau secretion and trans-synaptic lesion spreading (blue) may occur either directly from the axon due to loss of axonal MT integrity and secretion regulation, or indirectly from dendrites as the results of toxicity caused by somatodendritic tau accumulation.

Studies of tau biology raise the possibility of an alternative (or perhaps additional) mechanism to direct oligomerization in the cytosol. The catalysis of tau oligomerization by fatty acids is as consistent with a membrane-mediated assembly mechanism as it would be with a cytosol-only mechanism. More direct support for membrane-mediated NFT formation includes electron microscopic observations of membrane-associated PHFs in AD brain (126) and the experimental generation of tau filaments *in vitro* on the surfaces of anionic micelles (127). This occurs via tau intermediates containing β structures whose conformations are dependent on the presence of the anion and which are capable of seeding tau fibril formation. Tau that is not associated with MTs interacts with actin (18) and/or actin-associated proteins (128, 129) causing misprocessed tau to accumulate under the plasma membrane and in membrane-bound vesicles in cellular tauopathy models (19, 81, 105, 130, 131). This could plausibly result in tau endocytosis leading to vesicle-associated tau being returned to the perinuclear region via the retromer pathway, followed by NFT formation via membrane-templated oligomerization (125, 127). The Golgi apparatus and the autophagy-lysosome pathway are additional possible sources of tau-bearing vesicles that could contribute to NFTs. Abnormalities in Golgi structure have been identified both in association with tau overexpression (132) and with NFTs in AD brain (133). A recent study of PHF tau uptake in neuronal cultures suggested that endocytosed tau aggregates eventually form perinuclear aggresome-like deposits, which also suggests involvement of trans-Golgi “retromer” pathways in NFT formation (134). Similarly, the disruption of proteasome/macroautophagy (autophagy) mediated tau turnover is a prominent early element in pre-fibrillar changes in AD cytopathology (59), as evidenced by the polyubiquitination of tau in NFTs (135). Experimental disruption of lysosomal function via chloroquine administration induced lysosomal accumulation of tau aggregates (136), suggesting that disease-associated changes might contribute to NFT formation in AD via a similar mechanism. Similar patterns of somatodendritic tau distribution and associated signs of toxicity are seen in cortical pyramidal neurons with pre-fibrillar tau deposits or nascent NFTs (24, 113, 114, 137). These patterns suggest a role for vesicle-associated tau in both local tau cytotoxicity and in the generation of NFTs (81, 138). Examples are shown in Figure 2. In both cases, tau accumulates first in distal, membrane-rich dendritic structures (113, 139) and then at branch points and along dendritic shafts, where it is correlated with local MT loss, causing varicosities to appear in dendrites (113, 131). In the lamprey model, which is the only *in situ* tauopathy model from which high resolution localization information is available, these are associated with the accumulation of tau-bearing vesicles (and membrane-bound organelles such as mitochondria) at either end of the varicosity, suggesting that they are due to the failure of bidirectional MT mediated transport (Figure 1B). The accumulation of tau-containing vesicles at dendritic branch points is likely due to the shift in MT polarity patterns (140) typically found there, which induces the accumulation of vesicular organelles such as mitochondria. This vesicular build-up may be either the cause or consequence of MT-mediated transport failure (141–143).

### Tau toxicity mechanisms – are NFTs toxic?

The widely observed correlation between NFT distribution and neurodegeneration in nearly all tauopathies including AD (82, 83, 113, 114) has led to the widespread assumption that NFTs are an integral feature of tau neurotoxicity. Although toxic tau aggregates are notoriously difficult to generate in cell culture from wild-type tau isoforms (144), studies with hyperaggregating tau mutants have demonstrated that cleavage products are toxic when expressed in culture, with aggregate formation and apoptotic cell death occurring within 24–48 h of tau expression (145, 146). That said, it is becoming increasingly evident that NFTs may not be the agent driving neurotoxicity in whole animal tauopathy models. In *Drosophila*, expression of both mutant and wild-type human tau leads to AD-like pathology (late onset neurodegeneration, selective toxicity of cholinergic neurons) in the absence of NFTs (147). In addition, the long time courses (up to 20 years) proposed for NFT formation based on imaging data from AD brain (148–150) are not consistent with direct causality between NFTs and tau toxicity. While NFT growth is largely irreversible in inducible mouse tauopathy models, more dynamic aspects of tau toxicity are clearly reversible (151–154), suggesting that the mature NFT itself is much less toxic than the events associated with building it. Dendritic and axonal changes associated with tau accumulation and NFT formation in both transgenic mice (155) and AD brains (114, 156) appear to be correlated with abnormal mitochondrial distribution, which in turn recruits low ATP and Ca++ mediated toxicity mechanisms (144). Ca++ mediated tau toxicity is also suggested by the effects of specific tauopathy mutations on Ca++ channel properties (55) and high resolution correlations between localized secretion, MT loss, and accumulations of vesicular tau (81, 131) as illustrated in Figure 1. These findings and others have complicated our understanding of the relationship between NFT distribution and abundance and the actual toxicity mechanisms driving human neurodegenerative disease. Recent studies have also tended to dissociate degenerative cellular changes from NFT formation. It has even been suggested that large tau aggregates such as NFTs may serve a neuroprotective role (157, 158), preventing hyperphosphorylated tau from sequestering normal, MT-bound tau. Studies in other tauopathy models have also called into question the role played by hyperphosphorylation in the chain of events leading to degeneration; recent fly model studies (159) have suggested that hyperphosphorylation can be neuroprotective by blocking other aspects of tau toxicity, such as apoptotic changes associated with cell cycle re-entry (80, 160).

#### Oligomer-associated toxicity mechanisms

Tau oligomers have been the most widely proposed candidate for the toxic intermediate species in NFT biogenesis responsible for the correlation between neurofibrillary lesions and neurodegeneration in AD and non AD tauopathies (83). The toxicity of oligomeric tau is suggested by numerous correlative studies (161) and in particular with respect to the dynamic effects of tau aggregation; for instance, the concentration of tau multimers (162) but not large aggregates or monomers (95) in the brains of tauopathy mice are correlated with memory and cognitive deficits.

##### Oligomer-mediated membrane permeability changes

The structural similarity between amyloid proteins associated with neurodegeneration supports the existence of a common toxicity mechanism based on common properties of such proteins such as their propensity for oligomerization and close association with membrane. The toxicity of amyloid oligomers unrelated to neurodegenerative diseases suggests that a specific, shared conformation may be responsible, with toxicity being mediated by mitochondrial dysfunction associated with an increase in reactive oxygen species (141, 163). Oligomers of several different amyloids cause an increase in ion conductance across lipid bilayers (164) raising the possibility that they might alter or the permeability of the plasma membrane, resulting in increased internal [Ca++] and associated toxic changes. Both Ab and SNCA can form oligomeric structures that increase ion permeability of synthetic vesicular membranes and which may allow them to create pores within cell membranes (107). Leakage of cellular contents across the plasma membrane in SH-SY5Y cells was observed after the external application of tau oligomers (108), suggesting a similar toxicity mechanism for tau. These results collectively suggest that tau oligomers may mediate any of several toxicity mechanisms associated with Ca++ dysregulation or abnormal generation of reactive oxygen species. Such changes are typically seen with excitotoxicity and mitochondrial dysfunction, both of which appear to be important elements of AD-associated neurotoxicity (141). Studies in cellular tauopathy models showing a correlation between localized cytotoxicity, tau membrane association, and localized MT loss (81, 130, 165) are consistent with this (see examples in Figure 2), as is the presence at the plasma membrane of polyanionic molecules known to catalyze tau oligomer/filament formation such as HSPGs (30, 166) and RNA (123).

##### Oligomers as prions

The “prionlike” toxicity and propagation mechanisms recently proposed for tau may be considered as a special category of oligomer-mediated toxicity, if one makes the assumption that a prionlike misfolded tau conformation would be toxic and would propagate in a manner similar to that of the PrP itself. However, despite the considerable amount of research effort devoted to understanding the relationship between propagation and toxicity mechanisms of the PrP, this relationship remains quite unclear. Mutant forms of PrP that do not create large amounts of misfolded, pathogenic prion protein (PrP^Sc^) with high beta-sheet content in plaques have still been shown to generate CJD-like syndromes in mice (167). Moreover, interference with the normal GPI linkage that anchors PrP to the membrane can affect the ability of the mutant form to propagate interneuronally and generate a clinical syndrome but without affecting local cytotoxicity (168). Tau misprocessing appears to have a number of parallels to PrP toxicity in this regard; like PrP, tau oligomerization, and post-translational modifications that favor tau oligomer formation (e.g., hyperphosphorylation, truncation) are closely associated with toxicity. In the case of tau, it remains unclear whether higher-level oligomers and/or polymers propagate interneuronally in human tauopathies. Recent studies using murine tauopathy models have suggested that they are capable of this (98, 99, 169).

##### Disruption of protein turnover pathways

The dysfunction of proteasomal, autophagosomal, and lysosomal pathways with protein aggregate formation in tauopathy is a candidate for mediating tau toxicity as well as NFT formation, and may play an important role in the development of other tau containing cellular lesions, such as granulovacuolar degeneration [Ref. (170), reviewed in Ref. (171)] in which tau accumulations appear to be membrane associated. The polyubiquitinated state of NFT-tau suggests involvement of the ubiquitin-proteasome pathway (135). Additionally, the co-localization of tau aggregates with high concentrations of acid hydrolases in granulovascular degeneration (GVD) bodies in the hippocampal neurons of AD patients suggests that they may be the result of incomplete autophagy (172, 173). Lysosomal activation is also observed in cultured cells transfected with mutant tau and in mice expressing mutant tau transgenes (174). Inhibition of lysosomal proteases by tau misprocessing causes the accumulation of amphisome-resembling vacuoles in cultured neurons that are morphologically similar to those seen in AD brains. Inhibition of vesicular transport by tau, especially N-terminal tau fragments (175) may also prevent the fusion of autophagosomes and lysosomes (176), causing the retention of tau-bearing autosomes in axons and dendrites, where their accumulation may result in localized degeneration and even tau secretion [Figure 2, also see Ref. (81)]. One way that dysfunction of the autophagy-lysosomal machinery caused by tau aggregates may induce toxicity is enhancing tau oligomerization by the generation of hyperaggregating tau cleavage fragments due to the incomplete activity of lysosomal proteases. Cathepsin L has been shown to generate aggregation-prone fragments mutant but not wild-type tau via association with Lamp2A and Hsc70 on the cytosolic face of the lysosomal membrane (177). Tau-induced disruption of autophagy may also recruit synergistic effects related to the production of Abeta from APP, since PS1 is also necessary for autophagosome-lysosome fusion and lysosomal proteolysis (178). It should also be noted that the vesicle trafficking pathways associated with aggresome formation may also become abnormally involved in tauopathy as demonstrated by a recent study in which tau aggregates were endocytosed and then localized to perinuclear deposits (134).

#### Non-oligomer-mediated tau toxicity mechanisms

##### Receptor-mediated toxicity

An alternative to oligomer/aggregate associated mechanisms of tau toxicity to explain the selective vulnerability of neurons and spread of NFTs through synaptically connected regions in Alzheimer’s disease is toxicity in response to tau binding to extracellular receptors, especially those for synaptic transmitters such as glutamate and acetylcholine. Tau is toxic to neurons in culture when applied extracellularly (179), apparently via the generation of Ca++ fluxes via the activation of M1 and M3 muscarinic acetylcholine receptors (180), which bind tau with a greater affinity than acetylcholine. This is consistent with the preferential distribution of muscarinic acetylcholine receptors on entorhinal and hippocampal pyramidal neurons, and accounts for their vulnerability in early stages of Alzheimer’s disease (181). Interestingly, the dephosphorylation of secreted tau by tissue-non-specific alkaline phosphatase could potentiate its high-affinity binding to muscarinic receptors of nearby neurons (182), thus accounting for the “clustering” lesion spread patterns characteristically seen in tauopathies (90, 91, 183).

The generation of Ca++ fluxes via receptor-mediated toxicity need not directly involve aggregate or oligomer formation as a variety of MTBR− tau species appear to form multiple toxic fragments via Ca++ mediated activation of calpains and caspases in neuroblastoma cultures (109, 184) in at least some cases by NMDA receptor activation (109). A well studied fragment generated by calpain activity is a 17-kDa N-terminal fragment consisting of tau residues 45–230; this is toxic when applied to aged primary hippocampus cells and when expressed in the brains of transgenic *Drosophila* and in ABCs in the lamprey tauopathy model (142, 185, 186). The 17-kDa fragment is elevated in cortical neurons of AD and tauopathy patients that exhibit increased calpain activity (112) and is unusual among toxic tau species in its ability to produce neuritic pathology in neuroblastoma lines (185). However, it remains unclear if the 17-kDa fragment produced by calpain cleavage is a cause of neuronal death in tauopathy (187). Another N-terminal tau fragment with a molar mass of 20–22 kDa was found enriched specifically in the synaptosomes of AD brains in comparison to control brains (184). When overexpressed in primary rat neurons, an N-terminal tau fragment containing residues 26–44 impaired mitochondrial function and caused neuronal death (188). Downregulation of calpains in a tauopathy model of *Drosophila* was associated with decreased neurodegeneration (186). Both the tau N-terminal domain and fyn-tau interactions are associated with the mechanism of Ab toxicity that is mediated by the presence of tau (63, 74, 111), as are many fragments generated by similarly activated caspases (189, 190).

##### Can “prionic” and receptor-mediated N-terminal tau toxicity coexist?

The ability of tau to mediate Ab toxicity in the absence of the MTBR (111, 190) and the potential for NFTs to act as neuroprotective agents (157, 158, 191) raise questions about how large a role tau aggregate toxicity plays as a direct agent of neurodegeneration, at least in AD (112, 192). The links between toxic N-terminal tau fragments and excitotoxicity are consistent with the known vulnerability of cortical neurons with glutamatergic inputs and high levels of synaptic plasticity to AD (84) and the strong association between Ab mediated toxicity in early stage disease with synaptic dysfunction (193), suggesting that oligomer toxicity is not necessarily required for neurodegenerative disease pathogenesis. We recently pointed out that receptor-mediated toxicity mechanisms could account for neurofibrillary lesion propagation via Ca++ mediated activation of calpains and caspases, which could (at least in theory) generate both NFTs and secretable toxic fragments in downstream neurons, which could then repeat the cycle. Ironically, such a mechanism would technically fulfill the requirements of the original Prion Hypothesis, which makes no mention of templated misfolding (194). It thus seems safe to say that coexistence is not only possible but necessary given what we know (and don’t know) about how tau toxicity actually operates in neurodegenerative disease.

### Tau secretion

#### Cellular mechanisms of tau secretion

Tau secretion by neurons via multiple biologically distinct pathways has been demonstrated both in culture and *in situ* (105, 106, 195), despite its lack of a signal peptide and of lipidation or GPI anchor sites that would permit its secretion via the conventional ER/Golgi route. Tau resembles other aggregation-prone proteins with key roles in neurodegenerative disease (i.e., SNCA, PrP, and Ab) in that it is secreted at least in part via the exosome pathway (106, 196–198); however the full range of secretion routes and their significance to tauopathy pathogenesis remain unclear. Human tau phosphorylated at Thr181 (epitope AT270) is secreted in exosomes in culture and is also found in exosomes in the cerebrospinal fluid (CSF) of early stage AD patients (106). The early appearance of exosomal CSF tau in AD argues strongly in favor of CSF-tau biogenesis by active secretion rather than passive postmortem release, since it occurs at a stage (Braak Stage 3) when neurofibrillary degeneration is restricted to a small proportion of the brain (199). The N terminal of tau, which interacts with the plasma membrane and membrane-associated proteins, is required for secretion in culture and in an *in situ* lamprey model (195), where tau secretion occurred in two distinct patterns depending on the presence of the MTBR, a pattern consistent with the CSF-tau species observed in AD (200). In the lamprey model, N-terminal tau species lacking the MTBR become distributed in a diffuse, gradient-like pattern with secretion occurring from the soma, while full-length tau was secreted from the dendrites in discrete foci (195), where it set up much steeper tissue gradients, suggesting a role for interactions between the tau MTBR and extracellular matrix elements in the distribution of extracellular tau that could have relevance to interneuronal tau toxicity patterns and lesion spreading mechanisms (81). Interestingly, the secreted tau in the lamprey model is largely dephosphorylated, which is consistent with the phosphatase activity described by Diaz-Hernandez and co-workers (182), although no overt toxicity to non-expressing cells was observed. Secreted tau cleaved at the C terminal has been observed in cell culture (105), and in transgenic mouse models expressing human tau as well (201, 202). One of these studies suggested that cleavage at D421 as well as phosphorylation increased the rate of tau secretion (203), which is consistent with the low level oligomerization observed in exosomal tau purified from CSF samples (106).

The method(s) by which tau undergoes secretion remain elusive, although most evidence supports an unconventional secretion pathway resulting in the release of tau in membrane-bound vesicles (81, 106, 195, 203), where it is favored by the absence of the MTBR and/or the E2/E3 inserts in the secreted tau species (195). The failure of 4R2N tau to localize to and be secreted via exosomes is consistent with this as well (134), as are recent demonstrations that minute amounts of full-length, non-vesicle-associated tau can be released by induced forebrain iPS cells in culture (204). Such findings are consistent with a specific, non-universal release mechanism for tau that is associated with exosomes, as is the failure of an earlier study to detect exosomal tau secretion from healthy cortical neurons (205). Vesicle-free tau secretion has also been reported from neuroblastomas (106, 204) and may also occur in the lamprey tau secretion model (195). It thus remains possible that the presence of non-vesicular tau in the extracellular space in any model is due to post-secretion release of tau from exosomes or other microvesicles (105, 106, 195, 201, 202). Conversely, it could be that the presence of tau in exosomes may be due to the passive adherence of extracellular tau to exosomes that have already been released. This seems unlikely, given: (a) the restriction to E2− tau seen for exosomal tau and (b) the degree of interconnectivity of exosomal proteins associated with E2− overexpression via both functionally (Figure 2C) and via observed physical interactions (Figure 2C). Overexpression of tau in culture causes cells to secrete exosomes containing tau that has been phosphorylated at several proline-directed sites (106), possibly as a protective response to high concentrations of membrane-associated tau (206). Overall, it appears that tau may be secreted via multiple mechanisms from neurons in tauopathy, including microvesicle shedding, exosomal secretion via endocytosis and fusion with multivesicular bodies, and exophagy, a pathway involving the diversion of autophagosomes to exosomes (69, 81, 134, 207). Uptake mechanisms into trans-synaptic and adjacent cells have been characterized in even less detail that have tau secretion pathways. In the lamprey system, uptake has largely mirrored the “focal” and “diffuse” secretion mechanisms, with specificities for MTBR+ and tauopathy mutations and MTBR−, E2− tau respectively. A recent study of PHF tau uptake found evidence for perinuclear localization of tau in aggresome-like bodies (134), which is consistent with the scheme suggested in Figure 1. While the uptake of tau aggregates has been addressed by a number of recent studies directed at “prionlike” interneuronal transmission mechanisms via oligomeric tau species, the general mechanisms that might mediate tau uptake have not yet been characterized in detail in cell culture.

#### Interneuronal propagation of tau lesions and prions

Injection of brain extract from mutant human tau P301S transgenic mice into the brains of mice expressing wild-type human tau caused aggregation of wild-type tau in anatomically connected regions of the brain at a distance from the inoculation site (169). Sequential progression of tau aggregation (but not the transfer of individual tau molecules) between synaptically connected regions of the brain was later confirmed in mouse models in which human tau expression was spatially limited to synaptically connected areas of known vulnerability to neurofibrillary degeneration such as the entorhinal (98) and cingulate cortices (99). Further studies using antibodies specific to oligomerized tau have demonstrated that pre-fibrillar tau in AD brain is in fact oligomeric (208) and that tau oligomers derived from the brains of AD patients can recruit endogenous tau to oligomers both *in vitro* (97) and *in vivo* (96) and that intracerebral injection of oligomeric but not fibrillar wild-type tau caused aggregate formation from endogenous tau in synaptically connected but distant areas (96). Interestingly, regions receiving propagated tau in this study do not exhibit signs of tau toxicity, unlike SNCA, which did mediate toxicity as well as protein propagation via oligomer inoculation (100), a finding that illustrates the current ambiguous status of the “tauon” both as a toxicity agent and as a lesion spreading mechanism. In cell culture models, intercellular movement of misprocessed tau between cells and the apparent transfer of conformational alterations to endogenous tau has been more directly demonstrated. Endocytic tau uptake (verified using dextran co-localization) followed by fibril formation was observed in the proximity of labeled aggregates, suggesting recruitment of endogenous tau (92). Double immunolabeling (36) and fluorescence resonance energy transfer (37) has confirmed association of internalized tau with newly formed aggregates composed of endogenous tau. The uptake and axonal transport (retrograde and anterograde) of exogenous full-length tau aggregates has since been reported in differentiated primary neurons grown in Campenot chambers (209). Uptake was described as occurring via a process resembling bulk endocytosis, with the internalized tau associated with general lysosomal and endosomal markers (209). Common features of both cellular and mouse model observations of prionlike tau transfer include a specificity for oligomeric tau species and a requirement for the tau MTBR. The successful use of the 4R2N tau isoform for oligomer propagation (36) and the uptake of MTBR-only tau species (92) are obviously very different from the N-terminal specific E2− favored pattern observed for tau secretion in the lamprey model and in NB2A and M1C neuroblastoma cells (195). It should be noted that these differences reflect the tightly focused nature of the question being posed in the “prion” studies, i.e., whether oligomeric tau could be shown to propagate its oligomeric conformation between cells by recruiting endogenous tau in the recipient cell in the manner now established for misfolded PrP. This is very likely relevant to the one point of agreement between these approaches – that tauopathy mutations favor trans-synaptic tau transfer – since this type of movement may well be associated with oligomerization.

Recent investigations into interneuronal aspects of tauopathy pathogenesis have suffered from a bifurcated focus on either: (1) “prionlike” mechanisms of lesion spreading in terms of templated misfolding mechanisms derived from our understanding of prion diseases at either the whole animal or molecular level or (2) the morphological changes associated with tau secretion in cell autonomous models. These disparate approaches have (inevitably) been limited by: (a) their strict focus on molecular mechanism at the expense of cellular context and (b) exclusion of biochemical methodology in favor of spatial co-localization (respectively). These differences have dictated the choice of experimental models suited to the experimental approach taken (e.g., transgenic mice or lampreys), but have resulted in largely non-overlapping datasets devoid of a common context with which to consider them. This problem is exacerbated by a notable peculiarity of tau biology – its sensitivity to the terminally differentiated neuronal state. The inability of experimenters to induce tau-specific toxicity responses in cell lines (144) is likely due to the greater dependence of tau toxicity on axonal and synaptic functions relative to other “disease” proteins that are readily toxic in culture (210). While this has until now been a formidable obstruction to finding a broad-based approach to the characterization of mechanisms underlying tau toxicity and lesion spreading, it offers important hints for the direction of future studies on the topic – i.e., to focus on synaptic functions and developmental axon identity and guidance mechanisms to identify tauopathy-specific mechanisms, and to focus on aggregation-mediated mechanisms for identifying common themes of neurodegenerative disease pathogenesis.

### Is synaptic dysfunction the point at which tau oligomerization, autophagy disruption, tau secretion, and tau toxicity meet?

While the means by which tau becomes membrane associated and then diverted into unconventional secretion pathways is not yet clear, the interaction between tau and fyn kinase and the effects of that interaction on synaptic function is emerging as a key feature that might provide clarity. Fyn is rapidly localized to membrane raft domains by virtue of its double lipidation anchor sites (211). Fyn is capable of inducing exocytosis and/or endocytosis of membrane localized proteins (212, 213) and binds tau strongly via its proline rich region (19). Interactions between tau, fyn, and actin potentiate fyn-driven endocytosis of lipid raft markers flotillin-1 and flotillin-2 (129, 214). As shown in Figure 1, the Y18 residue of tau, a fyn substrate, is phosphorylated in distal dendritic vesicle accumulations localized to sites of focal tau secretion via microvesicles that contain both pY18 tau and endogenous fyn (81). This offers direct support for a mechanism by which fyn-activated endocytosis of raft domains containing tau results in localized tau secretion via unconventional pathways, one of which is exosomes. The phosphorylation of membrane and vesicle-associated tau by tauopathy-associated kinases (e.g., GSK3b, MARK kinases, and fyn) and their association with the exosome pathway is consistent with both receptor-mediated and oligomer-associated spreading and toxicity mechanisms, especially in the context of synaptic dysfunction.

#### Mislocalized tau and synaptotoxicity at glutamatergic synapses

Toxicity resulting from overexpression or extracellular application of N-terminal tau fragments in primary neurons appears to be caused at least in part by NMDA receptor-mediated Ca++ dysregulation (109), suggesting a potential link between Ca++ mediated transmitter release, tau secretion and plasticity-associated excitotoxicity and the dendritic accumulation of misprocessed tau at postsynaptic densities. A central role in this interaction may be mediated by fyn, particularly at highly plastic, glutamatergic synapses. NMDA receptor activity at glutamatergic synapses may mediate the tau-induced dendritic degeneration seen in lamprey ABCs, where fyn associates with and phosphorylates dendritically localized tau, resulting in both localized MT loss and tau secretion (81). Fyn-mediated activation of NMDA receptors normally appears to prevent excitotoxicity at glutamatergic synapses that exhibit LTP and LTD (77), but this mechanism may be vulnerable to disruption by abnormal perisynaptic tau accumulations (74, 215) and may account for the increased resistance to Ab-induced excitotoxicity observed in tau-reduced mice (216). Both the P301S (217) and P301L tauopathy mutations (75) in mice cause changes in hippocampal synaptic transmission and plasticity well before the onset of neuronal loss. This is consistent with the dependence of Ab toxicity on tau at synapses that exhibit plasticity (218) and suggests a modulatory role for both tau and Ab in synaptic plasticity that could increase sensitivity to excitotoxicity with the onset of neurodegenerative disease (216), accounting for the selective vulnerability of the hippocampus in AD. Similarly, mislocalized tau may also disrupt the AMPA receptor recycling associated with NMDA receptor activation via an increase in fyn-tau association and as a resulting increase in the rate of internalization of AMPA receptors on dendritic spines (77).

Chemical induction of LTD under conditions that prevent autophagosome/lysosome fusion causes LC3II-positive puncta to build up in dendritic spines, suggesting a link between AMPA recycling and autophagy (219). This could provide another point of access for misprocessed tau to toxicity and secretion mechanisms in AD, since inhibition of lysosomal acidification and/or the increased generation of Ab associated with *PSEN1* mutations associated with AD can increase Wnt signaling (220) as well as induce the collection of tau aggregates in lysosomes (221). Tau overexpression in both cell culture (136) and *in situ* tauopathy models (81) causes this as well, leading to the recruitment of both GSK3b, MARK and fyn kinases, which are all key mediators of Wnt pathway activity and tauopathy-associated tau kinases (222). This is consistent with the aberrant Wnt activity seen in AD (223) and the tau-dependent nature of Ab-induced toxicity via LTP in hippocampal synapses (218). Moreover, inhibition of GSK3b decreases the number of GR1 AMPA receptor subunits on the cell surface and increases their intracellular concentration (224). This pathway requires endocytosis of GSK3b into multivesicular bodies (225, 226). This provides potential links between the NMDA/fyn-mediated endocytosis of AMPA receptors seen with LTD (227–229) and the recruitment of tau into unconventional secretion pathways.

#### A unifying hypothesis

The tau accumulation at or near synapses that accompanies its mislocalization to dendrites and phosphorylation by GSK3b in tauopathy could easily potentiate the sequestration of both GSK3b and tau to late endosomes. This could result in increased sensitivity to LTP associated excitotoxicity (216) and the diversion of GSK3b (and tau) to exosomes, resulting in exosome-mediated tau secretion [(106) – also see Figure 2]. This mechanism is consistent with the increased exosome release (230) and tau secretion (231) seen with glutamate-induced AMPA and NMDA receptor activity and the decreased presence of postsynaptic AMPA receptors in the dendritic spines of P301 tauopathy mice (232). It is also consistent with the disruption of axonogenesis mechanisms in AD (such as those involving Wnt activity) and tau oligomerization, which may help recruit endosomal tau into exosomes (106, 233), as well as the observed distribution to and effects of tau in the dendrites in various model systems (78, 81, 234–236). Interestingly, we recently found that all of the major players in the above hypothetical scenario (Wnt markers, GSK3b, tau, fyn, MARK1, autophagy markers and NMDA, AMPA, and cholinergic receptor subunits) are enriched in exosomes released by neuroblastoma cells overexpressing full-length exon 2− (4R0N) human tau isoform (Figure 2A). Moreover, somatodendritic tau accumulation in the lamprey model appears to induce retrograde trans-synaptic localization toxicity accompanied by the cleavage and localization of LC3II to autophagosomes (Figure 2D). Such observations are consistent with a hypothesis in which misprocessed tau that has been dissociated from MTs and mislocalized to dendrites accumulates via fyn-dependent localization at postsynaptic densities and is then endocytosed and diverted to exosomes. The key element in this hypothesis is an endocytosis-associated mechanism that depends on tau-fyn interaction, induces tau oligomerization and is potentiated by both the activity of excitotoxicity-prone synapses and the progressive failure of tau to segregate normally to the axon in tauopathy pathogenesis, which increases the exposure of dendritic elements to tau, thereby amplifying perisynaptic tau toxicity [Ref. (69), also see Figure 2F]. The potential importance of changes in neuronal differentiation state to tau toxicity was recently underscored by a study of changes in gene regulation in two separate cohorts of early onset AD patients (237). They found that downregulated genes were preferentially associated with synaptic function and axonal identity, whereas upregulated genes were preferentially involved in the control of the cell cycle and in gene expression. We compared their results with those of an earlier study (238), which reported similar findings in LOAD patients. GO term analysis of a consensus set of down and upregulated proteins common to these studies (Figure 2E) are highly consistent with a failure of axonal and synaptic functions in the context of de-differentiation (possibly driven by cell cycle re-entry) producing a synergistic amplification of dendritic tau toxicity, a hypothesis outlined in more detail elsewhere (69). We therefore propose that tau secretion and toxicity via synapse-associated disruption of the TGN by mislocalized tau in dendrites may serve as a unifying idea that links multiple elements of tau pathobiology and that provides a context for better understanding the role of oligomerization in tau toxicity and lesion spreading.

## Conclusion

The increased appreciation of the significance of tau oligomerization in mediating tau neurotoxicity over the past decade must be counted as a major advance in our understanding of neurodegenerative disease. Increased understanding of the inherent toxicity of oligomers and the ability of at least some of them to induce the oligomerization of monomeric tau species, raises the possibility that the prionlike propagation of abnormal tau conformations may play a role in the spreading of tau lesions in the brain and that the complex process of oligomer and fibril formation may generate multiple toxic tau conformations that may have different mechanisms of action. However, it is not known if all oligomers are toxic and the toxicity mechanism of oligomeric tau remains obscure. Moreover, we have also learned that N-terminal tau fragments that are incapable of oligomerization can indeed be toxic and can mediate Ab-induced toxicity in the absence of the tau MTBR. Ironically, NFTs themselves do not appear to be toxic in many circumstances and may even be the result of a neuroprotective cell response that permits the long-term survival of neurons that bear them. While significant progress has been made on understanding disease-associated tau oligomerization and NFT biogenesis, it seems clear that not enough is yet known about the cellular mechanisms responsible for either tau secretion or tau toxicity for us to understand the relative significance of conformation specific or receptor-mediated tau cytopathology in models to the spreading of neurofibrillary tau lesions and neurodegeneration in human disease. Indeed, it remains possible that both N- and C-terminal fragments generated by incomplete tau proteolysis might act as synergistic prionlike vectors in a chain-reacting “Sorcerer’s Apprentice” scenario that could potentially account for sporadic tauopathy lesion patterns that are poorly explained by an oligomer mediated mechanism alone. Obviously, the operation of any mechanism generating multiple toxic N- and C-terminal tau fragments from a single molecule remains to be elucidated and cannot be evaluated seriously without far more knowledge of tau cell biology that we currently possess. Learning how phenomena such as exosomal (and non-exosomal) tau secretion and tau lesion propagation fit with tau-specific disease features linked to axonal identity and synaptic plasticity in more detail will mark a major step toward understanding the roles tau oligomers and other misprocessed tau species play in human disease.

## Conflict of Interest Statement

The authors declare that the research was conducted in the absence of any commercial or financial relationships that could be construed as a potential conflict of interest.

## Abbreviations

AD, Alzheimer’s disease; APP, amyloid precursor protein; CJD Creutzfeldt–Jacob disease; CNS, central nervous system; CSF, cerebrospinal fluid; ECF, extracellular fluid; ER, endoplasmic reticulum; FAD, familial AD; GA, Golgi apparatus; GO, gene ontology; HD, Huntington’s disease; HSPG, heparan sulfate proteoglycan; LOAD, late onset AD; MAP, microtubule associated protein; MT, microtubule; MTBR, microtubule binding repeat domain; NDD, neurodegenerative disease; NFT, neurofibrillary tangle; NT, neuropil thread; PD, Parkinson’s disease; PrP, prion protein; PrP^Sc^, misfolded, pathogenic prion protein; SNCA, alpha synuclein; TGN, trans-Golgi network.
